# Anticancer evaluation and molecular modeling of multi-targeted kinase inhibitors based pyrido[2,3-*d*]pyrimidine scaffold

**DOI:** 10.1080/14756366.2018.1437729

**Published:** 2018-02-27

**Authors:** Heba S. A. Elzahabi, Eman S. Nossier, Nagy M. Khalifa, Rania A. Alasfoury, May A. El-Manawaty

**Affiliations:** a Department of Pharmaceutical Chemistry, Faculty of Pharmacy (Girls), Al-Azhar University, Cairo, Egypt;; b Drug Exploration & Development Chair (DEDC), Department of Pharmaceutical Chemistry, College of Pharmacy, King Saud University, Riyadh, Saudi Arabia;; c Department of Pharmacognosy, Pharmaceutical Science Division, National Research Centre, Cairo, Egypt

**Keywords:** Pyrido[2,3-*d*]pyrimidine derivatives, anticancer activity, EGFR, CDK4/cyclin D1, molecular docking

## Abstract

An efficient synthesis of substituted pyrido[2,3-*d*]pyrimidines was carried out and evaluated for *in vitro* anticancer activity against five cancer cell lines, namely hepatic cancer (HepG-2), prostate cancer (PC-3), colon cancer (HCT-116), breast cancer (MCF-7), and lung cancer (A-549) cell lines. Regarding HepG-2, PC-3, HCT-116 cancer cell lines, 7-(4-chlorophenyl)-2-(3-methyl-5-oxo-2,3-dihydro-1*H*-pyrazol-1-yl)-5-(p-tolyl)- pyrido[2,3-*d*]pyrimidin-4(3*H*)-one **(5a)** exhibited strong, more potent anticancer (IC_50_: 0.3, 6.6 and 7 µM) relative to the standard doxorubicin (IC_50_: 0.6, 6.8 and 12.8 µM), respectively. Kinase inhibitory assessment of **5a** showed promising inhibitory activity against three kinases namely PDGFR β, EGFR, and CDK4/cyclin D1 at two concentrations 50 and 100 µM in single measurements. Further, a molecular docking study for compound **5a** was performed to verify the binding mode towards the EGFR and CDK4/cyclin D1 kinases.

## Graphical abstract

**Figure:**
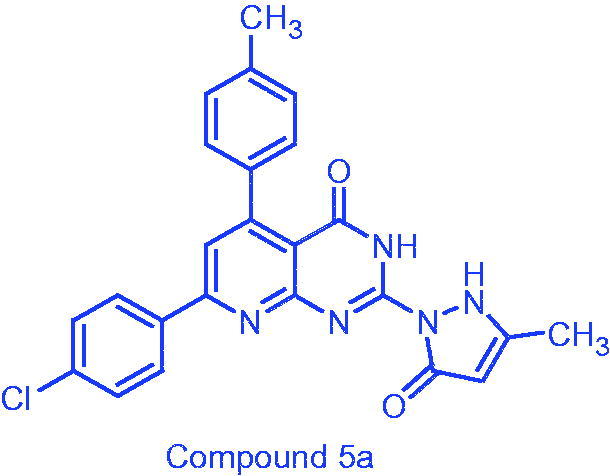

## Introduction

**Figure 1.**
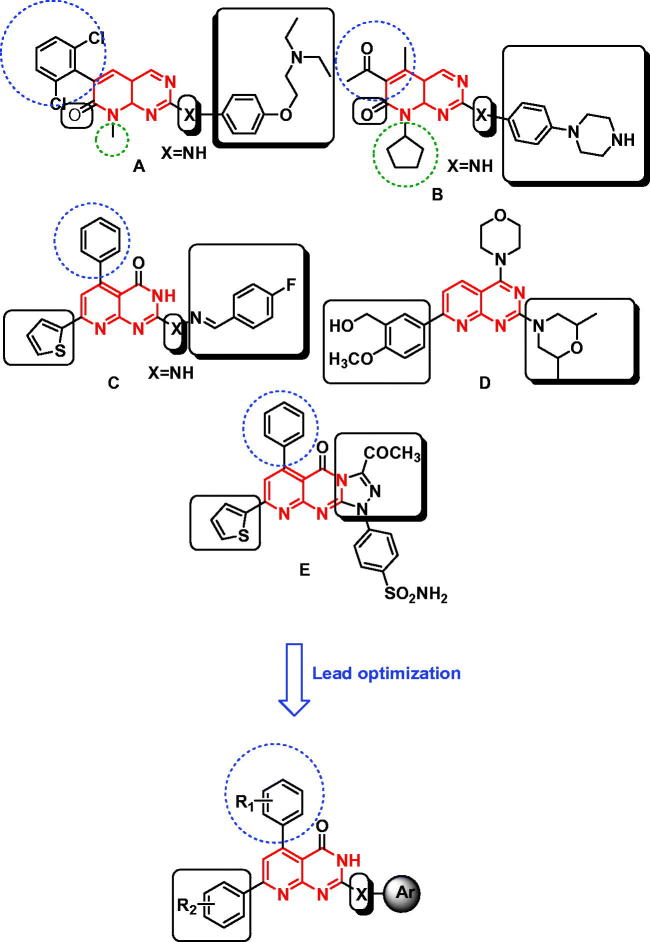
Reported and proposed pyrido[2,3-*d*]pyrimidine conjugates with anticancer and tyrosine kinase inhibitory activity.

Cancer is one of the leading causes of death in the world, characterized by the loss of control of cell proliferation, leading almost invariably to death, in untreated patients^1^ ^,^ ^2^. Chemotherapy, alone or in combination with surgery, is commonly the most efficient anti-cancer remedy. However, the use of available chemotherapeutics is limited mainly due to drug resistance and toxicities^3^. Developing resistance to chemotherapy belongs to many reasons like poor uptake of the drug, alternative metabolic paths, and increased production of the target protein, mutations that block the drug binding to its target or efflux systems that expel drugs from the cell^4–8^. So, combination of chemotherapies with different targets increases efficiency, antagonizes the resistance, and decreases toxicity as well. Traditional anticancer drugs work by disrupting the function of DNA. Some of these drugs may affect DNA directly or inhibit the enzymes controlling DNA synthesis. These drugs are mostly nonselective and having cytotoxicity to both cancer and normal cells^9^ ^,^ ^10^. The advances in molecular biology and genetics improve identification of molecular targets that are unique to cancer cells or overexpressed on them. The design of agents affecting these targets promises the development of more selective anticancer drugs with less toxic side effects^11^. Pyrido[2,3-*d*]pyrimidines were reported to display antitumor properties, which may be attributed to inhibition of different enzymes that involved in carcinogenesis cases. The prominent examples were pyrido[2,3-*d*]pyrimidines **A–E** that exhibited a potent inhibitory activity against various kinases, e.g. TKs, PI3K, and CDK4/6^12^ ^,^ ^13^ (Figure 1). Based on the structural features of the previous pyrido[2,3-*d*]pyrimidines, we aimed to prepare a new group of pyrido[2,3-*d*]pyrimidinone congeners, which were screened for their inhibitory activity against TKs, CDK4/6, and PI3K enzymes. Simultaneously, they were tested for their anticancer activity against cancer cells expressing the previous enzymes. Further, molecular modeling study was performed to explore the most appropriate binding modes of the most potent target compounds that matched ligands binding modes. The applied modeling program was Molecular Operating Environment (MOE®) 2008.10.

## Materials and methods

### Chemistry

Melting points by using electro thermal apparatus on open capillary tubes were recorded. IR spectra (KBr) were performed on a Shimadzu 435 IR Spectrophotometer (Shimadzu Bruker, Tokyo, Japan) (*ν* cm^−1^). ^1^H and ^13 ^C NMR NMR spectra were recorded on Varian Mercury VX-300 NMR 300 MHz spectrophotometer (Stoughton, USA) or on Agilent Technologies 400 MHz NMR spectrophotometer (Santa Clara, CA, USA) using DMSO-d6 as a solvent 300 MHz oron Agilent Technologies 400 MHz NMR spectrophotometer and the chemical shifts were estimated in *ppm*, relative to TMS as an internal standard. Physical data (C, H, and N) agreed with the proposed structures and obtained by using a Vario Elemental analyzer (Yamashitacho, Yokohama, Japan) (within ±0.4% of the theoretical values). Mass spectra were recorded on a DI-50 unit of Shimadzu GC/MS-QP 2010 plus Spectrometer (Livingston, West Lothian, UK) or on single quadrupole Mass Spectrometer ISQ LT (Thermo Scientific) (Austin, TX, USA).

### General procedure for the synthesis of 2,3-dihydro-2-thioxo-5,7-diarylpyrido[2,3-d]pyrimidin-4(1H)-one (3a–c, e–g)

2-Thioxopyrimidine **1** (1.43 g, 0.01 mol) and a series of α,β-unsaturated ketones **2a–g** (0.01 mol) in dry DMF (20 ml) was refluxed for 10–15 h and the reaction progress was monitored by TLC. The solid mass created on cooling was filtered and crystallized from (DMF) to give the title products.

#### 5-(2,4-Dichlorophenyl)-2,3-dihydro-2-thioxo-7-p-tolylpyrido[2,3-d]pyrimidin-4(1H)-one (3a)

Yield: 67%; mp: 309–311 °C; IR (KBr, cm^−1^) *ν*: 3402 (2NH), 1697 (C=O); ^1^HNMR (400 MHz, DMSO-d_6_): δ, 12.13 & 13.02 (2s, 2H, 2NH), 8.12 (d, 2H, *J* = 8.4 Hz), 7.92 (s, 1H, C-6 pyridine), 7.41 (s, 1H), 7.38 (d, 2H, *J* = 8.4 Hz), 7.33 (d, 2H, *J* = 8.4 Hz), 2.35 (s, 3H, CH_3_); MS: [*m/z* (%), 417 (0.84, M^+^+4), 415 (1.96, M^+^+2), 413 (2.28, M^+^), 119 (100)].

#### 7-(4-Bromophenyl)-2,3-dihydro-2-thioxo-5-p-tolylpyrido[2,3-d]pyrimidin-4(1H)-one (3b)

Yield: 88%; mp: 310–312 °C; IR (KBr, cm^−1^) *ν*: 3410 (2NH), 1701 (C=O); ^1^HNMR (400 MHz, DMSO-d_6_): δ12.13, 13.02 (2s, 2H, 2NH), 8.17 (d, 2H, *J* = 8 Hz), 7.92 (s, 1H, C-6 pyridine), 7.72 (d, 2H, *J* = 8.8 Hz), 7.41 (d, 2H, *J* = 8.8 Hz), 6.95 (d, 2H, *J* = 8.8 Hz), 2.06 (s, 3H, CH_3_); MS: [*m/z* (%), 425 (2.57, M^+^+2), 423 (2.04, M^+^), 180 (100)].

#### 7-(4-Chlorophenyl)-2,3-dihydro-2-thioxo-5-p-tolylpyrido[2,3-d]pyrimidin-4(1H)-one (3c)

Yield: 75%; mp: 302–304 °C; IR (KBr, cm^−1^) *ν*: 3394 (2NH), 1705 (C=O); ^1^HNMR (300 MHz, DMSO-d_6_): δ 12.25, 13.00 (2s, 2H, 2NH), 8.25 (d, 2H, *J* = 8.7 Hz), 7.94 (s, 1H, C-6 pyridine), 7.59 (d, 2H, *J* = 8.7 Hz), 7.34 (d, 2H, *J* = 9 Hz), 7.22 (d, 2H, *J* = 8.1 Hz), 2.37 (s, 3H, CH_3_); MS: [*m/z* (%), 381 (7.05, M^+^ +2), 379 (14.24, M^+^), 40 (100)].

#### 2,3-Dihydro-5-(4-methoxyphenyl)-2-thioxo-7-p-tolylpyrido[2,3-d]pyrimidin-4(1H)-one (3e)

Yield: 75%; mp: 361–363 °C; IR (KBr, cm^−1^) *ν*: 3421 (2NH), 1678 (C=O); ^1^HNMR (400 MHz, DMSO-d_6_): δ 12.28, 12.95 (2s, 2H, 2NH), 8.11 (d, 2H, *J* = 8 Hz), 7.55 (s, 1H, C-6 pyridine), 7.39 (d, 2H, *J* = 8.4 Hz), 7.38 (d, 2H, *J* = 8.8 Hz), 6.95 (d, 2H, *J* = 8.8 Hz), 3.82 (s, 3H, OCH_3_), 2.36 (s, 3H, CH_3_); ^13^CNMR (100 MHz, DMSO-d_6_): δ 175.71, 159.97, 153.79, 153.21, 141.19, 134.27, 130.88, 130.84, 129.93, 127.94, 119.23, 113.31, 108.02, 55.64, 21.42; MS: [*m/z* (%), 375 (14.85, M^+^), 91 (100)].

#### 7-(4-Chlorophenyl)-2,3-dihydro-5-(3,4,5-trimethoxyphenyl)-2-thioxopyrido[2,3-d]pyrimidin-4(1H)-one (3f)

Yield: 74%; mp: 306–308 °C; IR (KBr, cm^−1^) *ν*: 3402 (2NH), 1708 (C=O); ^1^HNMR (400 MHz, DMSO-d_6_): δ 12.31, 13.22 (2s, 2H, 2NH), 8.26 (d, 2H, *J* = 8 Hz), 7.93 (s, 1H, C-6 pyridine), 7.60 (d, 2H, *J* = 8.8 Hz), 6.76 (s, 2H), 3.70, 3.76 (2s, 9H, 3OCH_3_); MS: [*m/z* (%), 457 (0.1, M^+^ +2), 455 (1.6, M^+^), 95 (100)].

#### 2,3-Dihydro-5-(3,4,5-trimethoxyphenyl)-2-thioxo-7-p-tolylpyrido[2,3-d]pyrimidin-4-(1H)-one (3g)

Yield: 55%; mp: 318–320 °C; IR (KBr, cm^−1^) *ν*: 3421 (2NH), 1701 (C=O); ^1^HNMR (300 MHz, DMSO-d_6_): δ12.13, 12.98 (2s, 2H, 2NH), 8.15 (d, 2H, *J* = 8.4 Hz), 7.94 (s, 1H, C-6 pyridine), 7.34 (d, 2H, *J* = 8.1 Hz), 6.76 (s, 2H), 3.77, 3.72 (2s, 9H, 3OCH_3_), 2.38 (s, 3H, CH_3_); MS: [*m/z* (%), 435 (12.70, M^+^), 77.07 (100)].

### General procedure for the synthesis of 2-hydrazinyl -5,7-diarylpyrido[2,3-d]pyrimidin-4(3H)-one (4a–f)

Derivatives **3a**,**c–g** (0.004 mol) and hydrazine reagent 99% (3 ml, 0.006 mol) was refluxed in absolute ethyl alcohol (20 ml) for 10–15 h. On cooling, the residue created was filtered and purified from (DMF).

#### 5-(2,4-Dichlorophenyl)-2-hydrazinyl-7-p-tolylpyrido[2,3-d]pyrimidin-4(3H)-one (4a)

Yield: 27%; mp: 167–169 °C; IR (KBr, cm^−1^) *ν*: 3410, 3190 (2NH, NH_2_), 1678 (C=O); ^1^H NMR (400 MHz, DMSO-d_6_): δ 10.13 (1s, 1H, NH), 7.98 (s, 1H, C-6 pyridine), 7.55 (d, 2H, *J* = 8 Hz), 7.42 (d, 1H, *J* = 6.8 Hz), 7.38 (d, 2H, *J* = 10 Hz), 7.34 (d, 2H, *J* = 8 Hz), 6.17 (s, 1H, NH), 4.32 (s, 2H, NH_2_), 2.36 (s, 3H, CH_3_); MS: [*m/z* (%), 415 (1.69, M^+^+4), 413 (2.00, M^+^+2), 411 (4.86, M^+^)].

#### 7-(4-Chlorophenyl)-2-hydrazinyl-5-p-tolylpyrido[2,3-d]pyrimidin-4(3H)-one (4b)

Yield: 54%; mp: 233–234 °C; IR (KBr, cm^−1^) *ν*: 3421, 3190 (2NH, NH_2_), 1716 (C=O); ^1^HNMR (400 MHz, DMSO-d_6_): δ8.29, 8.28 (2s, 3H, NH, NH_2_), 8.19 (d, 2H, *J* = 8.8 Hz) , 8.08 (s, 1H, NH), 7.93 (s, 1H, C-6 pyridine), 7.53 (d, 2H, *J* = 8 Hz), 7.25 (d, 2H, *J* = 8 Hz), 7.17 (d, 2H, *J* = 8 Hz), 2.30 (s, 3H, CH_3_); MS: [*m/z* (%), 379 (2.32, M^+^ +2), 367 (100)].

#### 2-Hydrazinyl-5-(4-methoxyphenyl)-7-p-tolylpyrido[2,3-d]pyrimidin-4(3H)-one (4d)

Yield: 73%; mp: 260–262 °C; IR (KBr, cm^−1^) *ν*: 3425, 3290 (2NH, NH_2_), 1728 (C=O); ^1^H NMR (400 MHz, DMSO-d_6_): δ 10.17 (s, 1H, NH), 8.26 (s,1H, NH), 8.06 (d, 2H, *J* = 8 Hz), 7.30 (s, 1H, C-6 pyridine), 7.33 (d, 2H, *J* = 8.4 Hz), 7.29 (d, 2H, *J*= 8.8 Hz), 6.92 (d, 2H, *J* = 8.8 Hz), 4.20 (s, 2H, NH_2_), 3.79 (s, 3H, OCH_3_), 2.38 (s, 3H, CH_3_); MS: [*m/z* (%), 373 (100, M^+^)].

#### 7-(4-Chlorophenyl)-2-hydrazinyl-5-(3,4,5-trimethoxyphenyl)pyrido[2,3-d]pyrimidin-4-(3H)-one (4e)

Yield: 37%; mp: 279–281 °C; IR (KBr, cm^−1^) *ν*: 3421, 3111 (2NH, NH_2_), 1716 (C=O); ^1^H NMR (400 MHz, DMSO-d_6_): δ 10.03 (s, 2H, NH), 8.30 (d, 2H, *J* = 8 Hz), 7.93 (s, 1H, C-6 pyridine), 6.79 (d, 2H, *J* = 8 Hz), 7.62 (d, 2H, *J* = 8 Hz), 6.16 (s, 2H, NH_2_), 3.77, 3.75 (2s, 9H, 3OCH_3_); MS: [*m/z* (%), 454 (1.33, M^+^+1), 453 (3.52, M^+^), 77 (100)].

#### 2-Hydrazinyl-5-(3,4,5-trimethoxyphenyl)-7-p-tolylpyrido[2,3-d]pyrimidin-4(3H)-one (4f)

Yield: 68%; mp: 301–303 °C; IR (KBr, cm^−1^) *ν*: 3348, 3103 (2NH, NH_2_), 1716 (C=O); ^1^HNMR (400 MHz, DMSO-d_6_): δ 10.12 (s, 2H, 2NH), 8.19 (d, 2H, *J* = 8 Hz), 7.99 (s, 1H, C-6 pyridine), 7.36 (d, 2H, *J* = 8 Hz), 6.76 (d, 2H, *J* = 8 Hz), 6.12 (s, 2H, NH_2_), 3.76, 3.77 (2s, 9H, 3OCH_3_), 2.36 (s, 3H, CH_3_); MS: [*m/z* (%), 433 (1.49, M^+^), 115 (100)].

### General procedure for the synthesis of2–(3-methyl-5-oxo-2,3-dihydro-1H-pyrazol-1-yl)-5,7-diarylpyrido[2,3-d]pyrimidin-4(3H)-one (5a–d), 2-(3-amino-5-oxo-2,3-dihydro-1H-pyrazol-1-yl)-5,7-diarylpyrido[2,3-d]pyrimidin-4(3H)-one (6a–d) and 1-(4-oxo-5,7-diaryl-3,4-dihydropyrido[2,3-d]pyrimidin-2-yl)pyrazolidine-3,5-dione (7a–c)

2-Hydrazinyl derivatives (0.01 mol) and active methylene (ethyl acetoacetate, ethyl cyanoacetate, or diethylmalonate) (0.01 mol) was refluxed in glacial acetic acid for 4–6 h. After completion of the reaction, the precipitate created on pouring into ice-water was filtered and purified from (acetic acid) to give the title compounds **5a–d**, **6a–d** and **7a–c**, respectively.

#### 7-(4-Chlorophenyl)-2-(3-methyl-5-oxo-2,3-dihydro-1H-pyrazol-1-yl)-5-(p-tolyl)pyrido-[2,3-d]pyrimidin-4(3H)-one (5a)

Yield: 67%; mp: 385–387 °C; IR (KBr, cm^−1^) *ν*: 3422 (2NH), 1716 (2C=O); ^1^HNMR (400 MHz, DMSO-d_6_): δ 11.16, 11.66 (2s, 2H, 2NH), 8.21 (d, 2H), 8.18 (s, 1H, C-4 pyrazolone), 7.57 (d, 2H, *J* = 8 Hz), 7.50 (s, 1H, C-6 pyridine), 7.30 (d, 2H, *J* = 8 Hz), 7.19 (d, 2H, *J* = 8 Hz), 2.35 (s, 3H, CH_3_), 1.89 (s, 3H, CH_3_); ^13^CNMR (100 MHz, DMSO-d_6_): δ 172.44, 161.77, 157.81, 154.58, 153.98, 137.86, 136.26, 136.07,135.88, 129.63, 129.34, 129.09, 128.42, 118.39, 106.54, 21.50, 21.32; MS: [*m/z* (%), 446 (22, M^+^+2), 444 (50, M^+^), 186 (100)].

#### 2-(3-Methyl-5-oxo-2,3-dihydro-1H-pyrazol-1-yl)-5,7-di-p-tolylpyrido[2,3-d]pyrimidin-4-(3H)-one (5b)

Yield: 53%; mp: 342–344 °C; IR (KBr, cm^−1^) *ν*: 3429 (2NH), 1727, 1651 (2C=O); ^1^H NMR (400 MHz, DMSO-d_6_): δ 11.93, 13.59 (2s, 2H, 2NH), 8.28 (d, 2H, *J* = 8 Hz), 8.17 (s, 1H, C4-pyrazolone), 7.68 (s, 1H, C6-pyridine), 7.59 (d, 2H, *J* = 8 Hz), 7.37 (d, 2H, *J* = 8 Hz), 7.24 (d, 2H, *J* = 8 Hz), 2.35, 2.39 (2s, 6H, 2CH_3_), 1.88 (s, 3H, CH_3_); MS: [*m/z* (%), 423 (0.79, M^+^), 367 (100)].

#### 7-(4-Chlorophenyl)-2-(3-methyl-5-oxo-2,3-dihydro-1H-pyrazol-1-yl)-5-(3,4,5-trimethoxyphenyl)pyrido[2,3-d]pyrimidin-4(3H)-one (5c)

Yield: 42%; mp: 323–325 °C; IR (KBr, cm^−1^) *ν*: 3421(2NH), 1719 (2C=O); ^1^HNMR (400 MHz, DMSO-d_6_): δ 11.97, 13.71 (2s, 2H, 2NH), 8.31 (d, 2H, *J* = 8 Hz), 8.20 (s, 1H, C-4 pyrazolone), 7.78 (s, 1H, C-6 pyridine), 7.62 (d, 2H, *J* = 8 Hz), 6.78 (s, 2H), 3.73, 3.77 (2s, 9H, 3OCH_3_), 1.88 (s, 3H, CH_3_); ^13^CNMR (100 MHz, DMSO-d_6_): δ175.81, 162.13, 158.37, 155.58, 154.61, 152.70, 137.94, 136.27, 135.92, 134.87, 129.94, 129.49, 118.54, 106.85, 105.95, 60.50, 56.49; MS: [*m/z* (%), 521 (0.83, M^+^+2), 519 (3.84, M^+^), 376 (100)].

#### 2-(3-Methyl-5-oxo-2,3-dihydro-1H-pyrazol-1-yl)-7-(p-tolyl)-5-(3,4,5-trimethoxy phenyl)pyrido[2,3-d]pyrimidin-4(3H)-one (5d)

Yield: 43%; mp: 357–359 °C; IR (KBr, cm^−1^) *ν*: 3435 (2NH), 1719 (2C=O)); ^1^H NMR (400 MHz, DMSO-d_6_): δ 11.94, 13.67 (2s, 2H, 2NH), 8.20 (d, 2H), 8.18 (s,1H, C4-pyrazolone), 7.36 (d, 2H, *J* = 8 Hz), 7.72 (s, 1H, C6-pyridine), 6.76 (d, 2H, *J* = 8 Hz), 3.73, 3.77 (2s, 9H, 3OCH_3_), 2.38 (s, 3H, CH_3_), 1.88 (s, 3H, CH_3_); MS: [*m/z* (%), 500 (1.59, M^+^+1), 106 (100)].

#### 2-(3-Amino-5-oxo-2,3-dihydro-1H-pyrazol-1-yl)-7-(4-chlorophenyl)-5-(p-tolyl)pyrido [2,3-d]pyrimidin-4(3H)-one (6a)

Yield: 65%; mp: 382–384 °C; IR (KBr, cm^−1^) *ν*: 3430 (br, 2NH, NH_2_), 1726, 1644 (2C=O); ^1^HNMR (400 MHz, DMSO-d_6_): δ 13.90 (s, 2H, 2NH), 8.30 (d, 2H, *J* = 8 Hz), 8.18 (s, 1H, C-4 pyrazolone), 7.69 (s, 1H, C-6 pyridine), 7.61 (d, 2H, *J* = 8 Hz), 7.37 (d, 2H, *J* = 8 Hz), 7.25 (d, 2H, *J* = 8 Hz), 3.96 (s, 2H, NH_2_), 2.38 (s, 3H, CH_3_); MS: [*m/z* (%), 446 (22, M^+^+2), 444 (50, M^+^), 186 (100)].

#### 2-(3-Amino-5-oxo-2,3-dihydro-1H-pyrazol-1-yl)-5,7-di-p-tolylpyrido[2,3-d]pyrimidin-4-(3H)-one (6b)

Yield: 42%; mp: 380–382 °C; IR (KBr, cm^−1^) *ν*: 3429 (br, 2NH, NH_2_), 1727, 1680 (2C=O); ^1^H NMR (400 MHz, DMSO-d_6_): δ 12.48 (s, 2H, 2NH), 8.16 (d,2H, *J* = 8 Hz), 8.13 (s, 1H, C-4 pyrazolone), 7.80 (s, 1H, C-6 pyridine), 7.36 (d, 2H, *J* = 8 Hz), 7.32 (d, 2H, *J* = 8 Hz), 7.22 (d, 2H, *J* = 8 Hz), 3.97 (s, 2H, NH_2_), 2.37, 2.38 (2s, 6H, 2CH_3_); MS: [*m/z* (%), 424 (2, M^+^), 175 (100)].

#### 2-(3-Amino-5-oxo-2,3-dihydro-1H-pyrazol-1-yl)-7-(4-chlorophenyl)-5-(3,4,5-trimethoxyphenyl)pyrido[2,3-d]pyrimidin-4(3H)-one (6c)

Yield: 68%; mp: 339–341 °C; IR (KBr, cm^−1^) *ν*: 3433 (br, 2NH, NH_2_), 1726, 1678 (2C=O); ^1^HNMR (400 MHz, DMSO-d_6_): δ 13.68 (s, 2H, 2NH), 8.31 (d,2H), 8.20 (s, 1H, C-4 pyrazolone), 7.79 (s, 1H, C-6 pyridine), 7.60 (d, 2H), 6.78 (d, 2H), 3.96 (s, 2H, NH_2_), 3.79, 3.77 (2s, 9H, 3OCH_3_); MS: [*m/z* (%), 424 (2, M^+^), 175 (100)].

#### 2-(3-Amino-5-oxo-2,3-dihydro-1H-pyrazol-1-yl)-7-(p-tolyl)-5-(3,4,5-trimethoxy phenyl)pyrido[2,3-d]pyrimidin-4(3H)-one (6d)

Yield: 52%; mp: 350–352 °C; IR (KBr, cm^−1^) *ν*: 3390 (br, 2NH, NH_2_), 1716, 1675 (2C=O); ^1^H NMR (400 MHz, DMSO-d_6_): δ 11.46, 13.61 (2s, 2H, 2NH), 8.19 (d, 2H), 8.17 (s, 1H, C-4 pyrazolone), 7.72 (s, 1H, C-6 pyridine), 7.36 (d, 2H, *J* = 8 Hz), 6.78 (s, 2H), 3.96 (s, 2H, NH_2_), 3.73, 3.77 (2s, 9H, 3OCH_3_), 2.38 (s, 3H, CH_3_); ^13 ^C NMR (100 MHz, DMSO-d_6_): δ 171.74, 163.73, 159.72, 155.23, 154.45, 153.36, 152.68, 150.89, 144.52, 141.52, 141.35, 137.86, 135.06, 134.46, 130.03, 128.11, 118.25, 106.80, 60.49, 56.47, 21.44; MS: [*m/z* (%), 500 (M^+^, 0.7); 107 (100)].

#### 1-(7-(4-Chlorophenyl)-4-oxo-5-(p-tolyl)-3,4-dihydropyrido[2,3-d]pyrimidin-2-yl) pyrazolidine-3,5-dione (7a)

Yield: 23%; mp: 358–360 °C; IR (KBr, cm^−1^) *ν*: 3421 (2NH), 1716, 1730 (3C=O); ^1^HNMR (400 MHz, DMSO-d_6_): δ 12.39 (s, 2H, 2NH), 8.29 (d, 2H, *J* = 8 Hz), 7.89 (s, 1H, C-6 pyridine), 7.61 (d, 2H, *J* = 8 Hz), 7.33 (d, 2H, *J* = 8 Hz), 7.22 (d, 2H, *J* = 8 Hz), 2.95 (s, 2H, C-4 pyrazolidinone), 2.37 (s, 3H, CH_3_); MS: [*m/z* (%), 447 (14,M^+^+2), 445 (2,M^+^), 77 (100)].

#### 1-(7-(4-Chlorophenyl)-4-oxo-5-(3,4,5-trimethoxyphenyl)-3,4-dihydropyrido[2,3-d]pyrimidin-2-yl)pyrazolidine-3,5-dione (7b)

Yield: 66%; mp: 370–372 °C; IR (KBr, cm^−1^) *ν*: 3421 (2NH), 1732, 1660 (3C=O); ^1^HNMR (400 MHz, DMSO-d_6_): δ 13.95 (s, 2H, 2NH), 8.20 (d, 2H, *J* = 8 Hz), 7.70 (s, 1H, C-6 pyridine), 7.33 (d, 2H, *J* = 8 Hz), 6.65 (d, 2H, *J* = 8 Hz), 3.73, 3.77 (2s, 9H, 3OCH_3_), 2.95 (s, 2H, C4-pyrazolidinone); MS: [*m/z* (%), 521 (4, M^+^+2), 523 (0.8, M^+^), 95 (100)].

#### 1-(4-Oxo-7-(p-tolyl)-5-(3,4,5-trimethoxyphenyl)-3,4-dihydropyrido[2,3-d]pyrimidin-2-yl)pyrazolidine-3,5-dione (7c)

Yield: 34%; mp: 352–354 °C; IR (KBr, cm^−1^) *ν*: 3448 (2NH), 1732, 1690 (3C=O); ^1^HNMR (400 MHz, DMSO-d_6_): δ 12.92 (br s, 2H, 2NH), 8.31 (d, 2H, *J* = 8 Hz), 7.93 (s, 1H, C6-pyridine), 7.37 (d, 2H, *J* = 8 Hz), 6.78 (d, 2H, *J* = 8 Hz), 3.72, 3.76 (2s, 9H, OCH_3_), 2.94 (s, 2H, C-4 pyrazolidinone), 2.36 (s, 3H, CH_3_); ^13^CNMR (100 MHz, DMSO-d_6_): δ 170.45, 167.54, 167.16, 151.59, 147.78, 141.47, 134.15, 130.22, 128.01, 121.34, 106.85, 90.72, 60.47, 56.47; MS: [*m/z* (%), 501 (20, M^+^), 55 (100)].

### General procedure for the synthesis of2-(2-(aryl-2-ylmethylene)hydrazinyl)-5,7-diarylpyrido[2,3-d]pyrimidin-4(3H)-one (8a–f)

2-Hydrazinylpyrido[2,3-*d*]pyrimidines **4a**,**d** (0.01 mol) and aromatic aldehydes namely, benzaldehyde, 4-chlorobenzaldehyde, 4-anisaldehyde, or thiophene-2-carbaldehyde (0.02 mol) in acetic acid (10 ml) was refluxed for 4 h. The mixture was poured onto ice-water and the residue formed was filtered and purified from (acetic acid).

#### 7-(4-Chlorophenyl)-2-(2-(thiophen-2-ylmethylene)hydrazinyl)-5-(p-tolyl)pyrido[2,3-d]pyrimidin-4(3H)-one (8a)

Yield: 65%; mp: 360–362 °C; IR (KBr, cm^−1^) *ν*: 3421 (2NH), 1728 (C=O); ^1^HNMR (400 MHz, DMSO-d_6_): δ 13.5 (s, 2H, 2NH), 8.29 (d, 2H), 8.18 (s, 1H, N=CH), 7.70 (s, 1H, C-6 pyridine), 7.61–7.65 (m, 3H), 7.45 (d, 2H, *J* = 8 Hz), 7.32 (d, 2H, *J* = 12.3 Hz), 7.00 (d, 2H, *J* = 8 Hz), 2.38 (s, 3H, CH_3_); MS: [*m/z* (%) 473 (15, M^+^+2), 471 (10, M^+^), 43 (100)].

#### 2-(4-Chlorobenzylidene)hydrazinyl)-7-(4-chlorophenyl)-5-p-tolylpyrido[2,3-d]pyrimidin-4(3H)-one (8b)

Yield: 23%; mp: 366–368 °C; IR (KBr, cm^−1^) *ν*: 3423 (2NH), 1719 (C=O); ^1^H NMR (400 MHz, DMSO-d_6_): δ 13.71 (s, 2H, 2NH), 8.29 (d, 2H, *J* = 8 Hz), 8. 18 (s,1H, N=CH), 7.91 (d, 2H, *J* = 8 Hz), 7.59 (d, 2H, *J* = 8 Hz), 7.50 (s, 1H, C-6 pyridine), 7.39–7.34 (m, 4H), 7.25 (d, 2H, *J* = 8 Hz), 2.38 (s, 3H, CH_3_); MS: [*m/z* (%) 503 (2.14, M^+^+2), 499 (0.75, M^+^), 214 (100)].

#### 7-(4-Chlorophenyl)-2-(2-(4-methylbenzylidene)hydrazinyl)-5-(p-tolyl)pyrido[2,3-d]pyrimidin-4(3H)-one (8c)

Yield: 58%; mp: 375–377 °C; IR (KBr, cm^−1^) *ν*: 3427 (2NH), 1728 (C=O); ^1^H NMR (400 MHz, DMSO-d_6_): δ 13.5 (br s, 2NH), 8.28 (d, 2H, *J* = 8 Hz) , 8.18 (s, 1H, N=CH), 7.80 (d, 2H, *J* = 8 Hz), 7.60 (d, 2H, *J* = 8 Hz), 7.49 (s, 1H, C6-pyridine), 7.37 (d, 2H, *J* = 8 Hz), 7.33–7.23 (m, 4H), 2.28, 2.25 (2s, 6H, 2CH_3_); MS: [*m/z* (%) 481 (12, M^+^+2), 64 (100)].

#### 2-(2-Benzylidenehydrazinyl)-5–(4-methoxyphenyl)-7-(p-tolyl)pyrido[2,3-d]pyrimidin-4-(3H)-one (8d)

Yield: 53%; mp: 351–353 °C; IR (KBr, cm^−1^) *ν*: 3427 (2NH), 1728 (C=O); ^1^H NMR (400 MHz, DMSO-d_6_): δ 13.60 (s, 2H, 2NH), 8.13–8.18 (m, 3H), 7.64 (s, 1H, C-6 pyridine), 7.41–7.45 (m, 4H), 7.35 (d, 2H, *J* = 8 Hz), 6.97 (d, 2H, *J* = 8.8 Hz), 3.82 (s, 3H, OCH_3_), 2.37 (s, 3H, CH_3_); MS: [*m/z* (%) 461 (4, M^+^), 43 (100)].

#### 2-(4-Chlorobenzylidene)hydrazinyl)-5-(4-methoxyphenyl)-7-p-tolylquinazolin-4(3H)-one (8e)

Yield: 33%; mp: 361–363 °C; IR (KBr, cm^−1^) *ν*: 3422 (2NH), 1670 (C=O); ^1^H NMR (400 MHz, DMSO-d_6_): δ 11.72, 11.28 (2s, 2H, 2NH), 8.25 (s, 1H, N=CH), 8.11 (d, 2H, *J* = 8 Hz), 8.00 (d, 2H, *J* = 8 Hz), 7.43 (s, 1H, C-6 pyridine), 7.41–7.50 (m, 4H), 7.30 (d, 2H, *J* = 8 Hz), 6.94 (d, 2H, *J* = 8 Hz), 3.81 (s, 3H, OCH_3_), 2.36 (s, 3H, CH_3_); MS: [*m/z* (%) 497 (26, M^+^+2), 495 (84, M^+^), 384 (100)].

#### 5-(4-Methoxyphenyl)-2-(2-(4-methylbenzylidene)hydrazinyl)-7-(p-tolyl)pyrido[2,3-d]pyrimidin-4(3H)-one (8f)

Yield: 27%; mp: 380–382 °C; IR (KBr, cm^−1^) *ν*: 3421 (2NH), 1670 (C=O); ^1^H NMR (400 MHz, DMSO-d_6_): δ 11.82,11.73 (2s, 2H, 2NH), 8.10 (s, 1H, N=CH), 7.84 (d, 2H, *J* = 8 Hz), 7.37–7.45 (m, 4H), 7.33 (d, 2H, *J* = 8 Hz), 7.21 (d, 2H, *J* = 8 Hz), 6.95 (d, 2H, *J* = 8 Hz), 3.81 (s, 3H, OCH_3_), 2.35, 2.33 (s, 6H, 2CH_3_); ^13 ^C NMR (100 MHz, DMSO-d_6_): 161.20, 159.91, 140.73, 140.44, 135.38, 134.64, 133.87, 132.21, 130.66, 129.56, 129.85, 128.24, 127.98, 127.90, 127.70, 127.21, 117.49, 113.46, 113.24, 55.61, 21.51, 21.38; MS: [*m/z* (%) 475 (9, M^+^), 42 (100)].

## Biological evaluation

### *In-vitro* antitumor assay

Anticancer screening (*In vitro* bioassay on human cancer cell lines) was determined by the Bioassay-Cell Culture Laboratory, National Research Centre, Cairo, Egypt. It was adopted against five cancer cell lines (HepG2, PC-3, HCT116, MCF-7, and A549); doxorubicin was used as a reference standard according to a previously reported methods^14–16^.

### Kinase inhibition assay

The *in-vitro* enzyme inhibition determination for compound **5a** (which showed promising anticancer activity against HePG-2, PC-3, HCT-116 cancer cell lines in comparison with doxorubicin was carried out in KINEXUS Corporation, Vancouver, British Columbia, Canada. Kinexus has developed an open-access, on-line resource called DrugKiNET, www.drugkinet.ca. The evaluation performed profiling of the compound **5a** against a range of five protein kinases [(PDGFR beta, EGFR, CDK4/Cyclin D1, PI3K (p100b/p85a, PI3K (p100a/p85a)] according to a previously reported method^16^.

### Molecular modeling study

All the molecular modeling calculations and docking simulation studies were performed utilizing Molecular Operating Environment (MOE^®^) 2008.10^17^. The three-dimensional X-ray structures of EGFR (PDB code: 1M17)^18^ ^,^ ^19^ and CDK6 was used instead of CDK4 (PDB code: 2EUF)^20^ ^,^ ^21^ were obtained from the Protein Data Bank through the internet.

## Results and discussion

### Chemistry

New group of 5,7-diaryl pyrido[2,3-*d*]pyrimidinones **3a–g** were created through reaction of the starting precursor 2-mercapto-4-hydroxy-6-aminopyrimidine **1** with α, β unsaturated ketones **2a–g** in dry DMF according to the applied method reported for analogue **3d** ^22–24^. Compound **3c** as an example showed absorption band at 3394 cm^−1^ assigned for NH group in IR spectrum and three singlet signals at δ 7.94, 12.25, and 13.00 ppm attributed to C6 proton of pyridopyrimidine ring and 2NH protons in ^1^HNMR spectrum. Further, the MS revealed molecular ion peak at *m/z* 379 (M^+^) agreed with the molecular weight of the assigned structure. Also, ^13 ^C**-**NMR spectrum of **3e** analogue showed signal at 175.71 ppm for C=S group.

**Scheme 1.**
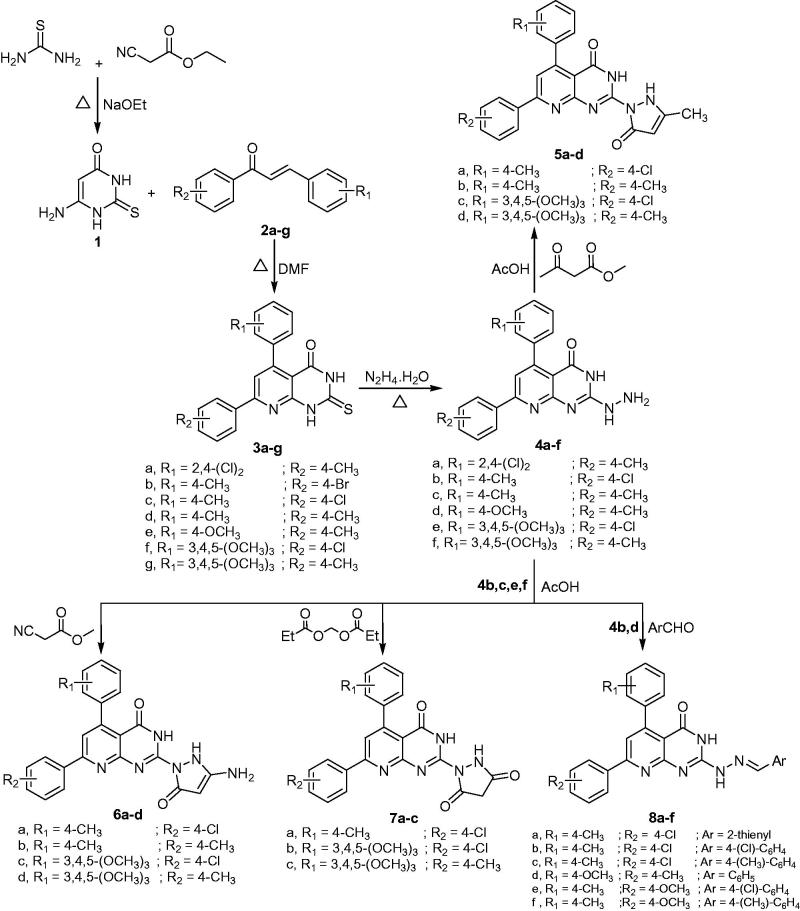
Synthesis of Pyrido[2,3-*d*]pyrimidine derivatives.

Nucleophilic attack^22–25^ of hydrazine hydrate on thioxo derivatives **3a**,**c–g** yielded the corresponding 2-hydrazinopyrido[2,3-*d*]pyrimidin-4(3*H*)-ones **4a–f** (Scheme 1). IR spectrum of compound **4a** showed existence of three absorption bands at 3410, 3190, and 1678 cm^−1^ corresponding to NH_2_, NH, and C=O groups, respectively. ^1^H NMR spectrum of **4d** revealed singlet signals at δ2.38, 3.79, 4.20, 8.26, and 10.17 ppm assignable for CH_3_, OCH_3_, NH_2,_ and two NH protons, respectively.

Derivatives **4 b**,**c**,**e**,**f** were condensed with active methylene compounds (ethylacetoacetate, ethylcyanoacetate, or diethylmalonate) in glacial acetic acid^26^ to give the corresponding three-substituted pyrazolone derivatives **5a–d**,** 6a–d**,** 7a–c**, respectively. Compounds **5a–d** were confirmed by the existence of an additional band at 1651 cm^−1^ for C=O group of the newly created pyrazolone nucleus in IR spectrum of compound **5b**. Additionally, the ^1^HNMR spectrum of **5a** showed signals at δ 1.89 and 8.18 ppm assignable for CH_3_ group and C4 proton of pyrazole moiety. In addition, ^13 ^C NMR spectra of **5a** revealed signals at δ 21.32, 106.54, 161.77, and 172.44 ppm assigned for CH_3_ group, C-4 of pyazole ring, C=O of pyridine ring and C=O of pyrazolone moiety, respectively.

On the other hand, **6a–d** derivatives displayed broad band at 3429 cm^−1^ for NH_2_ and two NH groups in IR spectrum of compound **6b**. Additionally, ^1^H NMR spectrum showed three singlet signals at δ 3.97, 8.13, and 12.48 ppm related to NH_2_ group, C4 of pyrazole moiety, and two NH groups, respectively. ^13^CNMR spectrum for compound **6d** showed signal at δ 21.44, 56.47, 60.49, and 106.80 ppm assigned for the carbon of the CH_3_, OCH_3,_ and C4 of the pyrazole moiety. The carbonyl signals were recorded at δ 163.37 and 171.74 ppm.

Compounds **7a–c** showed strong band for C=O group in pyrazole moiety at 1690 cm^−1^ in the IR spectrum of **7c** and presence of singlet signal equivalent to one proton at δ 2.94 ppm represents the CH_2_ protons of the pyrazolyl moiety in ^1^H NMR spectrum. ^13 ^C NMR spectra of compound **7c** showed signals at δ 56.47, 60.47, 90.72, 167.16, 167.54 and 170.45 ppm corresponding to the carbon of OCH_3_, C4 of pyrazole ring and three C=O carbons, respectively.

Condensation of the hydrazinyl derivatives **4 b,d** with various aromatic aldehydes^22^ ^,^ ^25^ afforded the corresponding 2-(2-(Aryl-2-ylmethylene) hydrazinyl)-5,7-diarylpyrido[2,3-*d*]pyrimidin-4(3*H*)-ones **8a–f**. Structures of compounds **8a–f** were confirmed by the presence of signals corresponding to the –N=CH proton at δ 8.10–8.25 ppm. ^13^CNMR spectrum for **8f** revealed signals at δ 21.38, 21.51, 55.61, 159.91, and 161.20 ppm assigned for CH_3_, OCH_3_, –NH = CH and C=O carbons, respectively. MS of compound **8 b** showed the molecular ion peak [M^+^] at *m/z* 499 and *m/z* 503 corresponding for the chlorine isotopes.

## Biological evaluation

### *Invitro* antitumor assay

All the synthesized compounds **3–8** were tested for their anticancer activity against hepatic cancer (HepG-2), prostate cancer (PC-3), colon cancer (HCT-116), breast cancer (MCF-7), and lung cancer (A-549) cell lines. Preliminary screening against the cancer cell lines was performed, using doxorubicin as a reference drug at doses of 100 μM. Variable results were recorded for the test compounds **3–8** (Table 1). Pyridopyrimidine derivatives that exhibited inhibitory activity >90% compared to doxorubicin were selected for IC_50_ and IC_90_ screening (Tables 2 and 3).

**Table 1.** Percentage of growth inhibition activity of compounds **3a–c**,**e–g**,** 4a**,**b**,** d–f**,** 5a–d**,** 6a–d**,** 7a–c**,** 8a–f** against HepG-2, PC-3, HCT-116, MCF-7, and A-549 cell lines at (100 μM) dose.

	Growth inhibition (%)				
Compound^a^	HepG-2	PC-3	HCT-116	MCF-7	A-549
**3a**	92	96	91	86	100
**3b**	26	53	30	41	81
**3c**	38	6	67	39	
**3e**	14	49	40	51	61
**3f**	69	92	44	86	98
**3g**	39	76	44	92	85
**4a**	90	100	90	37	99
**4b**	96	100	99	0	100
**4d**	96	100	96	68	99
**4e**	81	94	64	82	86
**4f**	24	66	40	0	78
**5a**	99	99	99	52	99
**5b**	35	23	60	54	34
**5c**	70	81	63	54	49
**5d**	99	98	93	93	95
**6a**	92	100	90	100	1
**6b**	63	4	69	34	45
**6c**	74	59	56	77	91
**6d**	85	99	88	88	100
**7a**	92	100	91	97	0
**7b**	80	75	52	73	84
**7c**	42	72	49	66	9
**8a**	64	98	66	83	78
**8b**	94	100	91	89	100
**8c**	99	99	93	96	83
**8d**	54	94	65	77	78
**8e**	35	40	57	23	32
**8f**	91.5	97	70	86	54
Doxorubicin^a^	100	100	100	91	100

a Concentrations of the test compounds and positive control (doxorubicin) were 100 μM.

**Table 2.** IC_50_ and IC_90_ of the test compounds **3a,f,g**, **4a,b,d,e**, **5a,d**, **6a–d,f** against HepG-2, PC-3 and HCT-116 cell lines.

			IC_50_/IC_90_ (μM)			
	HepG-2		PC-3		HCT-116	
Compound	IC_50_	IC_90_	IC_50_	IC_90_	IC_50_	IC_90_
**3a**	1.7 ± 0.56	19.27 ± 2.11	9.2 ± 0.9	17.5 ± 1.9	24 ± 3.2	48 ± 6
**3f**	–	–	55.69 ± 4	92.98 ± 9	–	–
**3g**	–	–	–	–	–	–
**4a**	13 ± 2.1	80.8 ± 4.6	14.96 ± 0.3	37.15 ± 2.1	38 ± 2.1	86 ± 5
**4b**	0.7 ± 0.09	17.6 ± 1	10.44 ± 1	18.46 ± 2	24 ± 2	–
**4d**	1.2 ± 0.5	19 ± 1.4	5.47 ± 0.4	15.57 ± 1.6	6.9 ± 0.9	21 ± 1.5
**4e**	–	–	32.52 ± 0.8	78.29 ± 4.9	–	
**5a**	0.3 ± 0.02	18.5 ± 2.5	6.6 ± 0.5	14.1 ± 0.8	7 ± 0.5	36 ± 2
**5d**	0.9 ± 0.06	–	26.7 ± 1.1	89 ± 4.2	5.9 ± 0.9	
**6a**	4.8 ± 1.03	21.6 ± 3.69	20.7 ± 1.3	79.9 ± 3.6	28 ± 1.2	53 ± 3.6
**6c**	–	–	–	–	–	
**6d**	–	–	7.9 ± 0.4	16 ± 2	–	
**7a**	0.3 ± 0.01	24 ± 2.5	24.7 ± 0.8	77.9 ± 4	25 ± 3.6	53 ± 5
**8a**	–	–	7.97 ± 0.2	53.35 ± 3.7	–	
**8b**	3.07 ± 1.4	22.3 ± 3.5	12 ± 0.9	23.5 ± 1.9	23 ± 1.8	60 ± 9.1
**8c**	1 ± 0.09	19.27 ± 1.1	11.65 ± 0.95	2.1 ± 0.3	28 ± 1.1	59 ± 8.9
**8d**		–	9.5 ± 0.99	85.82 ± 3.6	–	
	–					
**8f**	40.7 ± 8.9	84.6 ± 9.9	47.9 ± 2	91.6 ± 6.1	–	
Doxorubicin	0.6 ± 0.05	1.8 ± 0.2	6.8 ± 1.2	13.8 ± 0.8	12.8 ± 1	51.7 ± 0.7

IC_50_: Compound concentration required to inhibit the cell viability by 50%.

SEM: Standard error mean; each value is the mean of three values.

**Table 3.** IC_50_ and IC_90_ of the test compounds **3a,f,g**, **4a,b,d,e**, **5a,d**, **7a, 8a–d,f** against MCF-7and A549 cell lines.

	IC_50_/IC_90_ (μM)			
	MCF-7		A-549	
Compound	IC_50_	IC_90_	IC_50_	IC_90_
**3a**	–	-–	3.52 ± 0.5	25 ± 4
**3f**	–	–	28.79 ± 4	–
**3g**	30 ± 2.3	95 ± 11.2	–	
**4a**	–	–	–	–
**4b**	–	-–	10.94 ± 1.1	26.42 ± 3.1
**4d**	–	–	8.7 ± 0.9	24.8 ± 2
**4e**	–	–	–	–
**5a**	–	–	9.6 ± 1.1	27.8 ± 3.1
**6a**	21 ± 2.1	69 ± 6	–	–
**6c**	–	39.65 ± 2.1	83.44 ± 5.9	
**6d**	–	–	7.19 ± 1.6	37.57 ± 2.8
**7a**	33 ± 4.6	60 ± 9.7	–	–
**8a**	–	–	–	–
**8b**	–	–	14.6 ± 1.9	31.1 ± 2.5
**8c**	17 ± 2.1	69 ± 8.1	–	–
**8d**	–	–	–	–
**8f**	–	–	–	–
Doxorubicin	2.2 ± 3.1	5.2 ± 1.9	0.087 ± 0.9	0.35 ± 0.7

IC_50_: Compound concentration required to inhibit the cell viability by 50%; SEM Standard error mean; each value is the mean of three values.

Almost, hepatic cancer cell line (HepG2) showed remarkable sensitivity towards the test compounds **3–8** compared to doxorubicin. Pyrazolylpyrido[2,3-*d*]pyrimidines **5a** and **7a** were equipotent, possessing twice activity relative to doxorubicin (IC_50_ of 0.3 μM each and 0.6 μM respectively). Pyrazolyl analogue **5d** exhibited remarkable activity of IC_50_ 0.9 μM. Hydrazino precursor **4a** was almost equipotent to doxorubcin of IC_50_ 0.7 μM. Compounds **3a**, **4d** and **8c** displayed moderate activity of IC_50_ 1.7 μM, 1.2 μM, and 1 μM, respectively. Noticeably, the test compounds showed lower anticancer activity than doxorubicin, concerning IC_90_ measurement (Table 2).

Regarding the PC-3 cell line, hydrazino precursor **4d** and pyrazolyl analogue **5a** were more potent than doxorubicin as a reference standard (IC_50_5.47 μM, 6.6 μM, and 6.8 μM respectively). Also, pyrazolyl analogue **6d** as well as the hydrazone derivative **8a** displayed remarkable activity of IC_50_ 7.9 μM and 7.97 μM respectively. On the other hand, pyridopyrimidine derivatives **3a**, **4b**, **8 b–d** exhibited moderate activity with **IC_50_** ranged from 9.2 μM to 12 μM. Interestingly, IC_90_ showed promising anti-prostate cancer activity for pyrazolyl analogue **5a** versus doxorubicin (IC_90_ 14.1 μM and 13.8 μM, respectively). The rest of the test compounds showed IC_90_ ranged from moderate to poor activity compared to doxorubicin (Table 2).

Potent activity against HCT-116 cell line was recorded for three of the test compounds, e.g. 2-hydraino derivative **4d** and 3-methyl-5-oxo-pyrazolyl **5a**,**d**. They were almost twice the activity of doxorubicin exerting IC_50_ 6.9 μM, 7 μM, and 5.9 μM vs. 12.8 μM, respectively. Excepting pyridopyrimidines **4d** and **5a**,**d**, none of the test compounds showed high potency against HCT-116 colon cancer. Preferentially, the hydrazide derivative **4d** was also more potent than doxorubicin by two folds, exerting IC_90_ 21 μM vs. 51.7 μM, respectively. Simultaneously, compounds **3a** and **5a** exhibited higher activity than doxorubicin of IC_90_ 48 μM and 36 μM, respectively (Table 2). In contrary, breast and lung cancer cell lines exhibited remarkable resistance towards all of the test compounds **3–8** (Table 3).

### Structure-activity relationship

**Figure 2.**
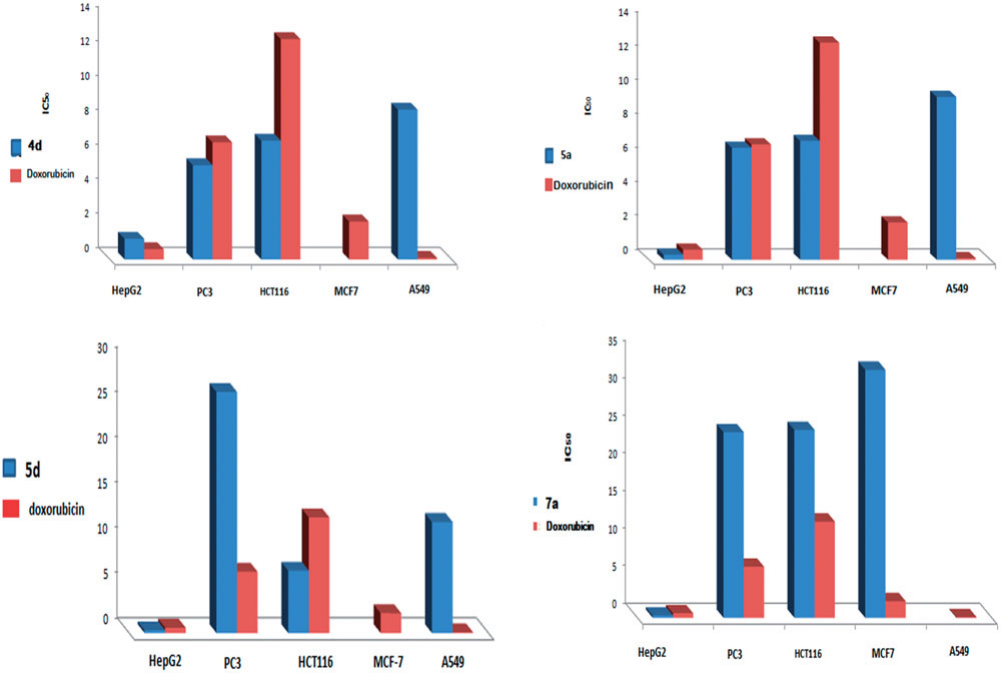
IC_50_ of compounds **4d, 5a, 5d, 7a** against HepG-2, PC-3, HCT-116, MCF-7, and A-549 cell lines.

In general, the structure–activity relationships of the screened products indicated that, existence of pyrazolyl moiety at C-2 of pyrido[2,3-*d*]pyrimidines **5a**,**d**,** 6d**, and **7a** derivatives afforded the maximum potency of anticancer activity. Noticeably, **5a** and **7a** shared the same pyridopyrimidine scaffold with (4-CH_3_-phenyl) and (4-chlorophenyl) at C-5 and C-7 respectively. Compound **5a** linked 3-methyl-5-oxopyrazolyl moiety showed broad anticancer effect against hepatic, prostate, and colon cancers. Replacing the methyl group of pyrazole moiety in **5a** with carbonyl group in **7a** afforded 3,5-dioxopyrazole where the activity profile was changed. Comparatively, it was retained against hepatic cancer but diminished against prostate and colon cancers in **7a**. Moreover, compounds **5d** and **6d** shared pyridopyrimidine scaffold with (3,4,5-trimethoxyphenyl) and (4-CH_3_-phenyl) at C-5 and C-7, respectively. Replacing methypyrazolone moiety in **5d** with aminopyrazolone in **6d** shifted the anticancer activity from anti-colon to anti-prostate cancer, respectively. Noticeably, the steric factor potentially affected the anticancer activity in **5a–d**. The data recorded remarkable decrease in the activity when (4-CH_3_-phenyl) group in **5a** was exchanged by (3,4,5-trimethoxyphenyl) in **5c**. Similarly, introduction of steric bulky group in **7b–c** diminished the anticancer activity. In contrary, steric factor did not affect the anticancer activity in **6a–d**, except **6d** derivative that carried the bulky (3,4,5-trimethoxyphenyl) group at C-5 (Figure 2).

Thioxo precursors **3a–c**,**e–g** displayed poor anticancer activity against all cancer cell lines. Anti-hepatic cancer effect was greatly increased upon converting thioxo group in **3c** to hydrazide moiety in **4b (**equipotent to doxorubicin**)**. Also, great records were displayed upon adopting the same replacement in **3e** to afford hydrazide analogue **4d**. The later was more potent than doxorubicin against prostate and colon cancers. Hydrophilic electron rich nature of the hydrazide moiety enabled the electronic factor to afford positive impact on the anticancer activity. Compounds **4b** and **4d** lacked their activity by extending the hydrazide moiety to benzylidene hydrazide in **8b**,**d**. Obviously, **8a–f** failed to record any potent activity against all cancer cell lines.

## Kinase inhibition assay

Upon cellular screening on HepG-2, PC-3, and HCT-116, compound **5a** exhibited higher anticancer activity in comparison with doxorubicin. So, it was subjected for *in vitro* inhibition assessment to measure its inhibitory activity against a panel of five different protein and lipid kinases. Two concentrations of **5a** were applied (50 μM and 100 μM) in single measurement compared to blank control.

Three of the tested enzymes are protein kinases, e.g. platelet-derived growth factor receptor (PDGFR) and epidermal growth factor receptor (EGFR) are tyrosine kinases. The third enzyme was cyclin-dependent kinase (CDK4/CyclinD) that phosphorylates serine/threonine residues. The remaining tested enzymes are lipid kinases that belong to phosphoinositide 3-kinases (PI3Ks), e.g. PI3K (p100b/p85a) and PI3K (p100a/p85a).

**Figure 3.**
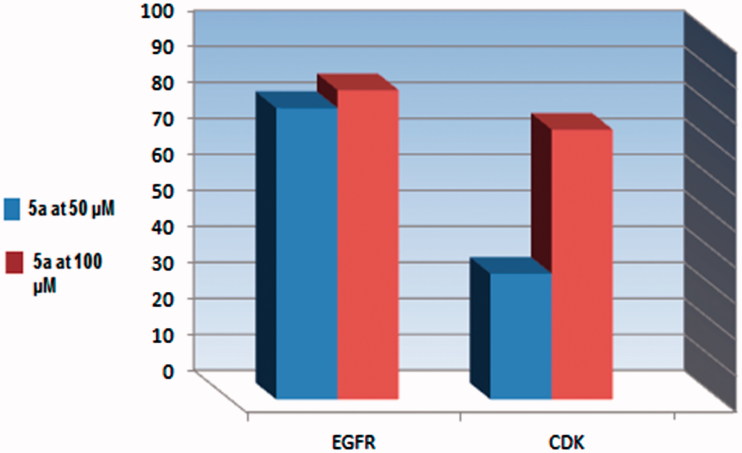
Percentage of kinases inhibition of compound **5a** against EGFR, CDK4 at (50 and 100 μM).

Maximum inhibitory percentage of **5a** was recorded (Table 4) against PDGFR beta of 82% and 94% at concentrations of 50 μM and 100 μM, respectively. Also, remarkable inhibitory percentage was exhibited against EGFR of 81% and 86% at the previously mentioned concentrations. On the other hand, **5a** at 50 μM recorded low inhibition percent against CDK4/Cyclin D1 of 35%. The inhibition of the previous enzyme was retrieved upon applying 100 μM of **5a** to 75%. As a conclusion, the inhibitory effect of **5a** against both PDGFR beta and EGFR was remarkable and directly proportional to the concentration. Interestingly, the inhibitory effect of **5a** toward PDGFR beta was more prominent and more concentration sensitive than EGFR. For example, increase the concentration of **5a** by two-fold from 50 μM to 100 μM afforded 10% increase of the activity against PDGFR. Concerning EGFR kinase, applying twice concentration of **5a** showed 5% increase of the activity. Although the effect of **5a** against CDK4/CyclinD1 was less prominent, but it was more concentration dependent. So, doubling the concentration of **5a** enhanced the inhibitory effect by almost two-folds (Table 4) (Figure 3). Unexpectedly, compound **5a** showed activation effect toward the two lipid kinases, PI3K (p100b/p85a) and PI3K (p100a/p85a) above the control experimental. The activation percentages were recorded in (Table 4). So, the potent anticancer activity of **5a** against HepG-2, PC-3, and HCT-116 could be attributed to its remarkable inhibitory activity against protein kinase enzymes.

**Table 4.** Percentage of kinases inhibition of compound **5a** at (50 and 100 μM).

	Compound **5a**	
	% Inhibition	
Kinase	50 μM	100 μM
CDK4/CyclinD1	−35	−75
EGFR	−81	−86
PDGFRβ	−82	−94
PI3K (p110a/p85a)	181	182
PI3K (p110b/p85a)	473	504

Negative (−) values: Inhibition of target activity by the compound; Positive (+) value: activation of target activity.

### Molecular docking results

Molecular docking technique represents the pattern of the interaction between a small molecule and a protein at the atomic level. This approach can explore the behavior of small molecules in the binding site of the target proteins. So, docking simulation was performed in this work using Molecular Operating Environment (MOE^®^)^17^ 2008.10. All the interaction energies and different calculations were automatically calculated.

Kinase inhibitory screening assay promoted compound **5a** that showed promising inhibitory activity against three kinases, namely PDGFR β, EGFR, and CDK4/Cyclin D1. The X-ray crystallography of PDGFR β structure was not fully resolved^27^. On the other hand, X-ray crystallography structure was reported for EGFR (pdb code: 1M17)^18^ ^,^ ^19^ with erlotinib. Simultaneously, the structure of CDK4/CyclinD1 with PD0332991 was reported (PDB ID: 2EUF)^20^ ^,^ ^21^. So the docking study was achieved for both EGFR and CDK4/Cyclin D1 kinases to predict the binding modes, affinities, and orientations of compound **5a** at the active sites of them.

### Docking study on EGFR

**Figure 4.**
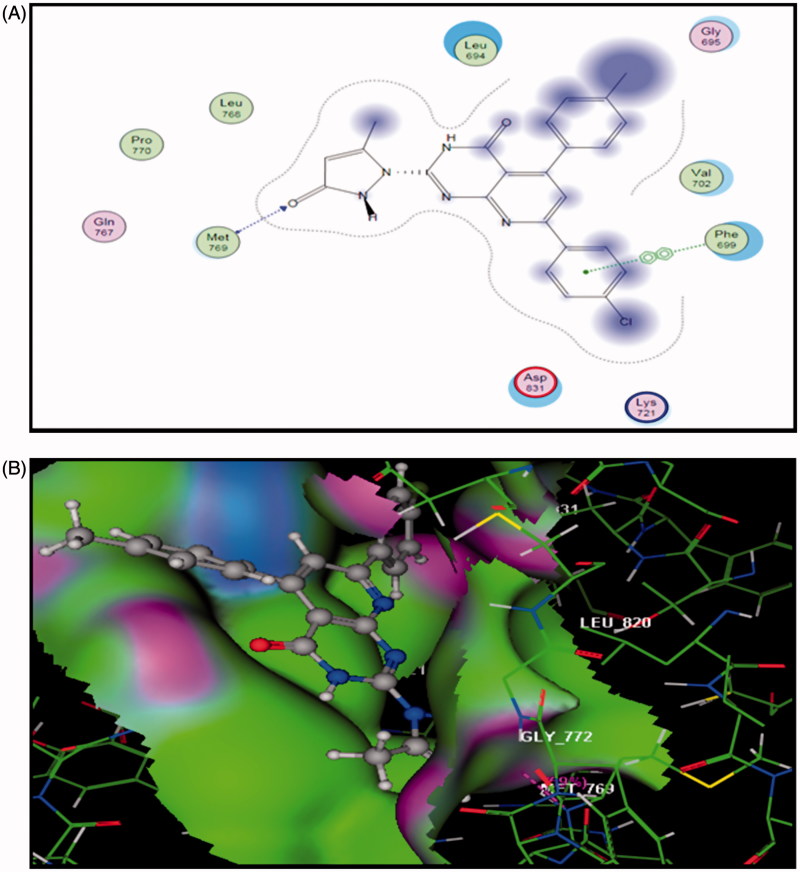
The proposed binding mode of compound **5a** docked in the active site of EGFR. A and B showing 2D and 3D ligand-receptor interactions (hydrogen bonds are illustrated as arrows; C atoms are colored gray, N blue, and O red).

Docking of **5a** into the active site of EGFR explored minimum binding energy comparable to the reference, erlotinib (Docking score= −7.95 and −5.35 Kcal/mol, respectively). The orientation of pyrido[2,3-*d*]pyrimidinone scaffold of **5a** was parallel to the hinge region due to the two bulky aryl rings. The scaffold posed in the adenine binding region of the ATP binding site. The pyrazolyl C=O carbon of **5a** formed a hydrogen bond acceptor (distance: 2.44 A^o^) with the backbone NH of **Met769** in the hinge region. Para-tolyl moiety shared hydrophobic interaction with **Gly695** and para-chlorophenyl ring formed arene-arene interaction with **Phe699** (Figure 4).

**Figure 5.**
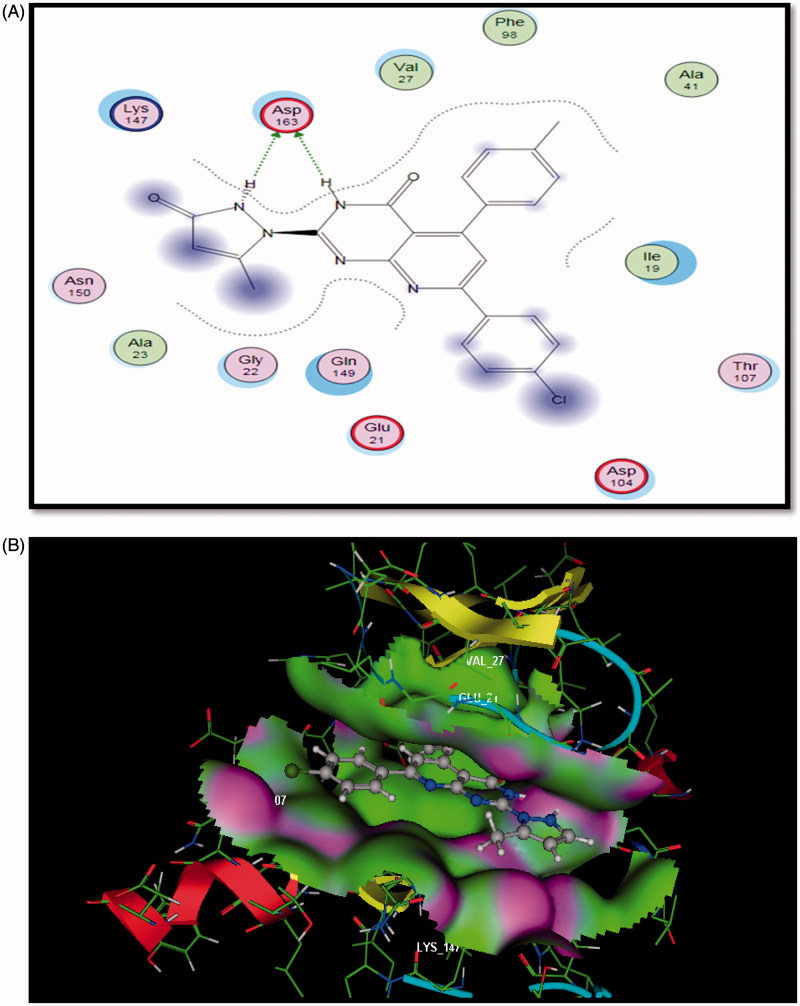
The proposed binding mode of compound **5a** docked in the active site of CDK6. A and B showing 2D and 3D ligand-receptor interactions (hydrogen bonds are illustrated as arrows; C atoms are colored gray, N blue, and O red.

### Docking study on CDK6

Compound **5a** bound in ATP binding pocket of CDK6 displayed good fitting and minimum binding energy as well as the reference PD0332991 (Docking score= -5.187 and -4.145, respectively). Also, it formed bidentate hydrogen bonding between N3 of pyridopyrimidine ring and N2 of pyrazolone moiety to the main chain amide of **Asp163** (distance =1.44, 1.43 A^o^). Moreover, para-chlorophenyl moiety projected on the opposite side, sequestered into a hydrophobic region (Figure 5).

So, docking simulation of compound **5a** into kinase domain of EGFR and CDK6 postulated the vital role of both pyrido[2,3-*d*]pyrimidinone scaffold and the side chain substituent. Both moieties involved in binding mode interaction. Binding pose covered different macromolecular interactions, e.g. H-bonding, arene-arene, and hydrophobic interactions. Compound **5a** could be anticipated to bind efficiently to the ATP binding site of EGFR compared to erlotinib. Furthermore, similar results were predicted for the binding pose of CDK6 with both PD0332991 and compound **5a**.

## Conclusions

A novel group of substituted pyrido[2,3-*d*]pyrimidinones **3**–**8**, were synthesized and evaluated for *in vitro* anticancer activity. There was potent growth inhibitory activity against HepG-2, PC-3, and HCT-116 cell lines and weak activity against MCF-7 and A-549 cancer cells in comparison to doxorubicin. Regarding HepG-2 cell line, compounds **5a, 7a** were more potent (IC_50_= 0.3 µM) than doxorubicin. Also, compound **4 b** was nearly equipotent (IC_50_= 0.7 µM) to doxorubicin (IC_50_= 0.6 µM). Upon cellular screening on PC-3, compounds **4d, 5a** exhibited higher activity than doxorubicin (IC_50_= 5.47, 6.6, 6.8 µM respectively). For HCT-116 cell line, **4d, 5a,** and **5d** derivatives showed two-folds the activity of doxorubicin (IC_50_= 6.9, 7, 5.9, and 12.8 µM, respectively). The highly potent anticancer compound **5a** exhibited promising inhibitory activity against PDGFR β, EGFR, and CDK4/Cyclin D1 kinases at two concentrations of 50 μM and 100 μM in single measurement. Furthermore, the molecular docking study for compound **5a** into the ATP binding site of EGFR and CDK6 explored an efficient binding as that of erlotinib and PD0332991, respectively.
